# A Bayesian generative model for learning semantic hierarchies

**DOI:** 10.3389/fpsyg.2014.00417

**Published:** 2014-05-20

**Authors:** Roni Mittelman, Min Sun, Benjamin Kuipers, Silvio Savarese

**Affiliations:** ^1^Department of Electrical Engineering and Computer Science, University of MichiganAnn Arbor, MI, USA; ^2^Department of Computer Science, University of WashingtonSeattle, WA, USA; ^3^Department of Computer Science, Stanford UniversityStanford, CA, USA

**Keywords:** Bayesian models of cognition, non-parametric Bayes, hierarchical clustering, Bayesian inference, semantics

## Abstract

Building fine-grained visual recognition systems that are capable of recognizing tens of thousands of categories, has received much attention in recent years. The well known semantic hierarchical structure of categories and concepts, has been shown to provide a key prior which allows for optimal predictions. The hierarchical organization of various domains and concepts has been subject to extensive research, and led to the development of the WordNet domains hierarchy (Fellbaum, 1998), which was also used to organize the images in the ImageNet (Deng et al., 2009) dataset, in which the category count approaches the human capacity. Still, for the human visual system, the form of the hierarchy must be discovered with minimal use of supervision or innate knowledge. In this work, we propose a new Bayesian generative model for learning such domain hierarchies, based on semantic input. Our model is motivated by the super-subordinate organization of domain labels and concepts that characterizes WordNet, and accounts for several important challenges: maintaining context information when progressing deeper into the hierarchy, learning a coherent semantic concept for each node, and modeling uncertainty in the perception process.

## 1. Introduction

There has been mounting evidence in recent years for the role that Bayesian probabilistic computations play both in the behavioral and the neural circuit layers of cognition (Chater et al., 2006; Steyvers et al., 2006; Tenenbaum et al., 2006; Fiser et al., 2010). In the behavioral layer, the assessments made by humans regarding everyday phenomena have been demonstrated to conform to those produced by a Bayesian calculation, integrating all the perception related uncertainty, as well as the prior knowledge, to produce an optimal prediction (Griffiths and Tenenbaum, 2006). In the neural circuit layer, hierarchical Bayesian generative models are gaining acceptance as the underlying mechanism for describing the neural computation process (Lee and Mumford, 2003; George and Hawkins, 2009). The Bayesian perspective has also been shown to allow for the learning of the appropriate structural forms (Kemp and Tenenbaum, 2008) for different cognitive problems. Structural forms are a prerequisite for making useful deductions, for example, in order to predict the number of days left until summer starts again we must first identify the cyclical pattern of the seasons. Similarly, object hierarchies provide the necessary structural form for object recognition. Such hierarchies organize different concepts and entities based on their semantic association and level of abstraction, and are central for fusing top-down and bottom-up information and making judicious deductions (e.g., if an entity is recognized to be a lion, and the hierarchy categorizes the lion as being dangerous, we could deduce that we had better take cover).

When considering visual recognition, the most basic questions relate to the form of object representation. A widely held belief, which has also been corroborated by fMRI experiments (Edelman et al., 1998), is that different objects are represented in a conceptual space where the dimensions are the responses of neurons. Semantically similar objects elicit responses which are geometrically closer in the conceptual space. Another observation of the geometrical model is that major categories, such as animals, contain smaller clusters such as faces and body parts (Mur et al., 2013). This is consistent with the hierarchical structural form for object recognition.

Although computer vision research generally proceeds independently from the cognitive sciences, in recent years themes such as semantic feature spaces and category hierarchies have become very influential in addressing many computer vision problems. The semantic concept space, discussed in the previous passage, has been emulated in the computer vision community through the use of attributes (Ferrari and Zisserman, 2007; Farhadi et al., 2009; Lampert et al., 2009; Dhar et al., 2011; Parikh and Grauman, 2011b). Attributes are detectors that are trained to predict the existence or absence of semantic concepts such as an eye, furry, or horizontally oriented. By employing several attribute detectors, each object can be represented as a point in the attribute space. Semantic hierarchies have become important in the field of fine-grained visual recognition, which aims at building systems which are capable of recognizing tens of thousands of categories, approaching the human capacity. The main use for such hierarchies has been to speed up (Griffin and Perona, 2008; Bart et al., 2011; Gao and Koller, 2011), and boost the accuracy (Marszałek and Schmid, 2007; Zweig and Weinshall, 2007; Kim et al., 2011) of object recognition systems. In Deng et al. (2012), a classifier was allowed to make predictions at various levels of abstraction. A Cocker Spaniel could be classified as a dog or an animal, depending on a compromise between specificity and accuracy. This illustrates the integration of different sources of uncertainty and prior knowledge, that is underlined in the Bayesian cognitive approach. A key element is the semantic hierarchy, which summarizes the coarse to fine relationship between the different categories and concepts at different levels of semantic granularity. Another use for semantic hierarchies, has been as a tool that simplifies the search and retrieval of images from large collections (Li et al., 2010).

Most of the methods that have been considered in the computer vision community for learning the semantic feature space and category hierarchies, rely on human intervention. The most straightforward approach for discovering semantic attributes is to query a domain expert. Other options include mining text and image data sampled from the Internet to automatically discover semantic concepts(Berg et al., 2010), or using a “human in the loop” strategy, in which human intervention is used to identify whether a discriminatively learned mid-level feature is also semantically meaningful (Kovashka et al., 2011; Parikh and Grauman, 2011a; Duan et al., 2012; Biswas and Parikh, 2013). Many computer vision algorithms that require the use of a category hierarchy, rely on a human specified taxonomic organization, such as the WordNet domains hierarchy (Fellbaum, 1998), which organizes a set of domain labels into a tree structure.

However, when children learn to identify objects, they construct both the concept space as well as the hierarchical object representation with minimal outside intervention. Simply through observation and interaction with different objects, they can identify the semantic similarities between many categories, and organize them in the appropriate hierarchical structure. This observation raises the question which computational models can be used to describe the learning processes of the concept space and the semantic hierarchy? When a child plays with his toys, he discovers basic regularities which are common to many of the examples that he observes and touches: flatness, roundness, box shaped, ball shaped, nose, mouth, etc. Therefore, learning the concept space corresponds to learning a mapping from the low-level sensory input, to each of these identified semantic properties. Recently, deep learning methods have been successful in learning mid-level feature representations that capture greater semantic content as compared to standard low-level image features. These approaches typically rely on techniques such as deep Boltzmann machines (Salakhutdinov and Hinton, 2009a; Salakhutdinov et al., 2011), restricted Boltzmann machines (RBMs) (Smolensky, 1986), and convolutional neural networks (LeCun et al., 1989; Krizhevsky et al., 2012). Deep learning methods learn a set of hidden units that can be used to describe the concept space, and have been shown to capture recognizable semantic content as well as the geometrical properties (Salakhutdinov and Hinton, 2009b) of the semantic feature space. Convolutional RBMs (Lee et al., 2011) have been successfully used to discover semantic concepts such as the wheels and windows of a car without using any form of supervision, purely based on sensory input of real valued image pixels. Convolutional neural networks have also been shown to provide a highly semantic mid-level representation when trained on very large datasets (Girshick et al., 2014). Using a weak form of supervision, provided by the category labels, semantic concepts such as “furry” and “snout” have been discovered using a RBM with a bag-of-visual-words based representation (Mittelman et al., 2013).

Unsupervised learning of hierarchies has been commonly addressed in the natural language processing context, where a large set of documents is used to learn a hierarchical structure in which semantically similar documents are assigned to nearby nodes. One example is the nested Chinese restaurant process (NCRP) (Blei et al., 2003a), which is a non-parametric Bayesian model that builds on the latent Dirichlet allocation (LDA) (Blei et al., 2003b; Griffiths and Steyvers, 2004). The LDA represents each document using a set of mixing proportions over topics. Each topic is represented by a multinomial distribution over the vocabulary, that captures the typical words that are associated with every topic. The NCRP assigns a unique topic to each node in the tree, such that each document is associated with a different path in the tree. The NCRP has also been used for learning visual hierarchies based on low-level image features and a bag-of-visual-words representation (Bart et al., 2011). However, since in contrast to text, low-level image features capture very little semantic content, the learned hierarchies do not display the geometric property of the concept space in which semantically similar categories are also assigned to nodes which are closer in the hierarchy (Li et al., 2010).

Since semantic hierarchies have been incorporated into many computer vision algorithms, in this work we are interested in developing a computational model that could describe how such hierarchies are formed. Bayesian models have become an important tool for describing cognitive processes, and therefore we propose a Bayesian generative model that learns a semantic hierarchy based on observations of objects in a concept space in which objects are represented as binary attribute vectors. Since the semantic distance between categories in WordNet has been shown to be correlated with the recognition difficulty (Deng et al., 2010) of computer vision algorithms, we would like the learned hierarchy to imitate several properties which characterize WordNet. Most importantly, WordNet organizes different objects and concepts into a set of complementary domain labels which follow a super-subordinate relationship. This allows the human knowledge to be organized in a single taxonomy of domains. Similarly, our learned hierarchy associates different attribute labels with each node, which effectively describe appropriate domain labels, and follows the super-subordinate semantic relationship. In the following section, we discuss the main properties of WordNet, as well as the importance of domain information when tackling visual recognition problems.

## 2. Wordnet domains hierarchy

The WordNet domains hierarchy organizes a set of 164 domain labels (Bentivogli et al., 2004) in a tree structure, which follows a super-subordinate relationship. More general concepts are linked to increasingly more specific ones, for example, since a car is a form of a transportation vehicle, the domain label “transportation” is the parent of the domain label “car.” Categories and concepts are grouped into sets of semantically equivalent words, which are known as “synsets,” and are assigned to the appropriate nodes which describe their semantics most accurately. Since many words have several meanings, depending on the context of the sentence in which they are used, different words may belong to several synsets [e.g., the word “bank” may relate to the domain “economy,” but it may also refer to other domains such as geography or architecture (Magnini et al., 2002b)]. The choice of domain labels, and the form of their organization, was designed such that each domain label has explicit and exclusive semantic interpretation, with similar granularity at each level. Furthermore, the hierarchy provides a complete representation of all human knowledge.

One of the main motivation factors of the developers of WordNet was the hypothesis that domain information is necessary in order to achieve semantic coherency in linguistic texts. Since different words can belong to several synsets, WordNet provides a powerful tool which can be used to identify the correct meaning of each word, and has been successfully applied for word sense disambiguation (Magnini et al., 2002a). A similar argument may help explain the underlying hierarchical organization of the concept space in which objects are represented, which also displays grouping based on a super-subordinate semantic relationships. As many attributes are shared by different categories, the context provided by the domain information allows for coherent interpretation of the object. For example, hands, eyes, and nose, are common to both people and monkeys, and therefore have to be disambiguated in order to coherently identify an entity as a person or a monkey. Object recognition experiments using a very large category count, have reported that the recognition difficulty is correlated with the semantic distance in the WordNet hierarchy (Deng et al., 2010). This supports the hypothesis that domain information is important for the object classification task.

The ImageNet dataset is a collection of more than 10,000,000 images with more than 10,000 categories, that are arranged in a hierarchical structure which is based on the WordNet domains hierarchy. Object recognition experiments performed using ImageNet have revealed that when considering recognition with a number of classes that is near the human capacity, WordNet can be used to classify categories in a varying degree of specificity. For example, a Golden Retriever can also be classified as a dog or an animal. All these outcomes are correct (although not equally favorable), and should entail a smaller penalty as compared to an outright misclassification, when designing a classifier.

## 3. A bayesian generative model for learning domain hierarchies

Since semantic hierarchies have found important use in many computer vision problems, we are interested in developing a Bayesian generative model that could describe the means by which the human visual system learns a similar hierarchy. Bayesian generative models have been gaining popularity as a means of describing many cognitive processes, and therefore offer a suitable building block for this purpose. Our proposed model, to which we refer as the attribute tree process, learns a domain hierarchy in an unsupervised fashion, based on a set of training images. We assume that the semantic concept space is described using binary feature vectors, where each component describes the existence or absence of some semantic property, to which we refer as an attribute. Some of these semantic concepts may be very general, such as “living things,” “transportation,” etc., while others are more specific such as “dog,” “car,” “leg,” etc. The hierarchical organization of these semantic concepts should capture the super-subordinate relationship that characterizes WordNet. Our generative model faces the following challenges: (a) maintaining the context information, (b) maintaining a coherent semantic interpretation for each node, and (c) modeling the uncertainty in the attribute observations (e.g., if the attribute “furry” is not active for an instance of the category “dog,” we would still like the instance to be assigned to an appropriate node in the hierarchy).

Learning the domain hierarchy requires us to learn the tree structure, and to associate a subset of the attribute pool to each node in the hierarchy. The selected attributes are used to describe the semantic concept which is associated with each node. The Bayesian framework allows us to infer both of these based on a training set, by specifying a probabilistic model which relates the node assignment of each data instance, to the semantic content associated with each node. Another critical issue, is to promote preference for a simple explanation of the data (“occam's razor”), which in our case corresponds to tree structures with few nodes. In order to learn the tree structure and assign each data instance to the appropriate node, we use a non-parametric Bayesian prior which is known as the tree-structured stick-breaking process (TSSBP) (Adams et al., 2010). The TSSBP is an infinite mixture model, where each mixture element is in one-to-one correspondence with a single node in an infinitely deep and infinitely branching tree structure. The mixture elements are formed by interleaving two stick-breaking processes (Ishwaran and James, 2001), which promote the formation of tree structures where only few nodes are associated with non-negligible mixture weights. The prior distribution for assigning an instance to a node, follows a multinomial distribution over the infinitely many nodes, with the TSSBP's mixture weights.

Our generative model assumes that given the node assignments of all the data instances, all the data instances are statistically independent. We use the following notation to describe the joint probability distribution of out model. We denote the observed binary attribute vectors using x_*i*_ ∈ ℝ^*D*^, *i* ∈ {1, …, *N*}, where *D* denotes the number of attributes, and *N* is the size of the training set. The assignment of an instance *i* to a node is denoted by *z*_*i*_ ∈ 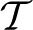, where 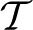 denotes the set of node indicators. The node parameters associated with node ϵ ∈ 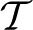 are denoted by θ_ϵ_. The joint probability distribution function is obtained using the Bayes rule:

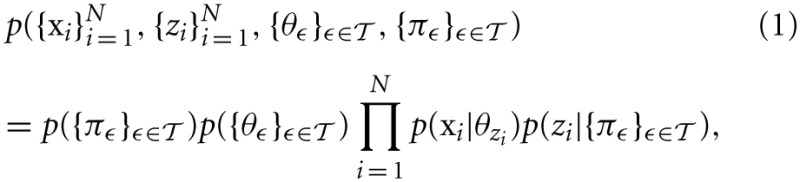



were *p*({π_ϵ_}_ϵ ∈
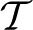
_), *p*({θ_ϵ_}_ϵ ∈
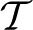
_) are the prior probability distributions for the tree structure, and for the node parameters, respectively. The conditional probability distributions *p*(x_*i*_|θ_*zi*_), *p*(*z*_*i*_|{π_ϵ_}_ϵ ∈
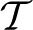
_), provide the likelihoods of an observation x_*i*_ given its assignment to node *z*_*i*_, and the likelihood an instance being assigned to node *z*_*i*_ given the TSSBP parameters.

**Figure 1:**
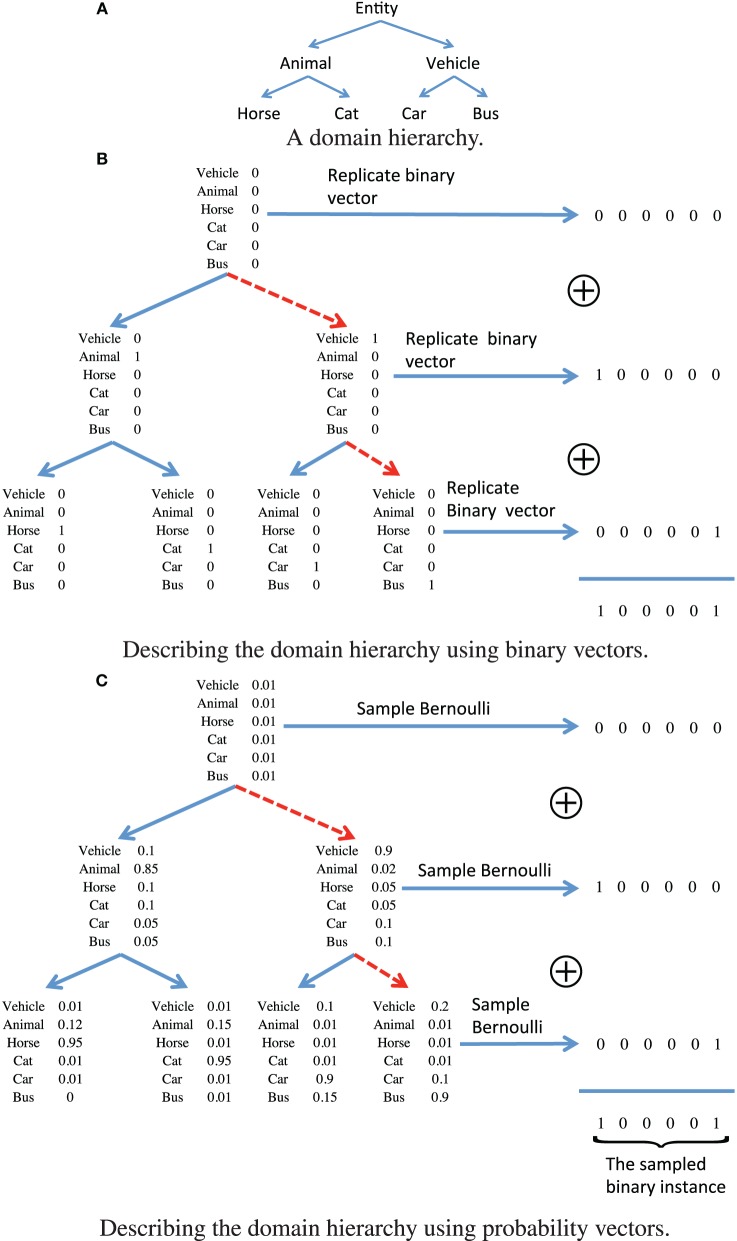
**An illustrative example of representing the (A) domain hierarchy using (B) binary vectors, and (C) using a relaxed probabilistic interpretation**.

Learning the domain hierarchy therefore corresponds to inferring the node parameters {θ_ϵ_}_ϵ ∈
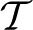
_. By providing a prior distribution for θ_ϵ_ for each ϵ ∈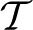, and the form of the conditional distribution *p*(x_*i*_|θ_*zi*_) which describes the likelihood of assigning training sample x_*i*_ to node *z*_*i*_, learning the node parameters can be achieved using Markov chain Monte-Carlo (MCMC) methods. Each data instance describes a subset of attributes, corresponding to various levels of semantic granularity. The domain hierarchy decomposes the binary instance vectors into a set of node parameters which correspond to standard basis elements, such that more general attributes are associated to nodes that are closer to the root node, and vice versa. This is demonstrated in Figure 1B for the domain hierarchy shown in Figure 1A. The data instance for a vector that is assigned to the node attached to the red dashed path in Figure 1B, is obtained using a logical or operation over all the node parameters associated with each of the nodes along the path. This form could be used to describe the conditional distribution *p*(x_*i*_|θ_*zi*_), however, it implies that all the data instances assigned to the same node must have the same set of attributes. In order to provide a probabilistic substitute to this hard association rule, we propose to relax this hard decision approach, such that each node parameter vector θ_ϵ_, ϵ ∈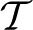 is a real valued vector of probabilities. The attributes associated with an instance assigned to each node are now described in a probabilistic framework, which is illustrated in Figure 1C for the node associated with the path described using the red dashed lines. For each node along the path, we first draw from a Bernoulli distribution with the corresponding node parameters, and then aggregate the binary vectors using a logical or operation. The probabilistic variation has important consequences when considering the variability of attributes observed in common images. For example, we may not observe the legs of a person in a scene as they may be occluded by a desk, however, we would still like that image to be assigned to a node in the hierarchy that is associated with the “person” category. In order to model additional uncertainty factors, we also flip the binary vectors that are generated according to the model that is illustrated in Figure 1C, with some attribute dependent probability ω^(*d*)^, *d* = 1, …, *D*, where *d* denotes the number of attributes. This also allows for weighting the different semantic concepts based on their reliability and importance. More important and reliably detected concepts should have a lower probability of being flipped, and vice versa.

By construction, the attribute tree process which is described above and is illustrated in Figure 1C, maintains the context information. Furthermore, it also accounts for the uncertainty in the observations since it is described using probabilistic tools. The remaining challenge is therefore to verify that the semantic concepts that are associated with each of the nodes are coherent. We argue that a necessary ingredient for this purpose, is to promote sparsity of the node parameters vector θ_ϵ_ for each ϵ ∈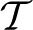. This ensures that each node is associated with a minimal subset of attributes which are necessary to describe its content, and avoid the assignment of unrelated semantic concepts to the same node. Moreover, since the generative process which is illustrated in Figure 1C, implies that attributes that are associated with any node are also going to be associated with all of its descendants, the sparsity constraint at node ϵ only needs to be applied to attributes which have not been associated with any ancestor of node ϵ. Such a form of sparsity constraint can be realized by choosing the prior for the node parameters to follow a finite approximation to a hierarchical Beta-Bernoulli process (Paisley and Carin, 2009). Specifically, for the node parameters at the root node we have that

(2) $$\theta_{0}^{\left(d\right)}\sim \text{Beta}\left(a/D,b(D-1)/D\right),d=1,\ldots ,D,$$

and the parameters in the other nodes follow

(3) $$\theta_{\epsilon }^{\left(d\right)}\sim \text{Beta}\left(c^{\left(d\right)}\theta_{\text{Pa}(\epsilon )}^{\left(d\right)},c^{\left(d\right)}\left(1-\theta_{\text{Pa}(\epsilon )}^{\left(d\right)}\right)\right),d=1,\ldots ,D,$$

where Pa(ϵ) denotes the parent of node ϵ, and where *a*, *b*, and *c*^(*d*)^, *d* = 1, …, *D* are positive scalar parameters, and where *D* denotes the number of attributes. The form of the prior for the node parameter vector at the root node that is given in Equation (2) promotes sparsity, whereas the prior for all the other node parameters that is given in Equation (3) promotes similarity to the parameter vector of the parent node. Therefore, this choice promotes sparsity for all the attributes which have not been already associated with an ancestor node.

In summary, in this section we proposed a Bayesian generative model that learns a hierarchical organization of semantic concepts at different levels of abstraction, such that a super-subordinate relationship is satisfied. To this end, we relaxed the hard assignments of attributes to nodes in the hierarchy, such that the assignment assumes a probabilistic form. This allows for better modeling of the uncertainty of the association between attributes and categories, and allows for efficient inference and learning using Markov chain Monte-Carlo methods. In order to promote coherent semantic interpretation of each node, we incorporated a hierarchical sparsity prior which encourages the selection of a minimal subset of necessary semantic concepts to be associated with each node. Modeling the *a priori* preference for trees with fewer nodes was achieved by incorporating the TSSBP, which is a non-parametric Bayesian prior for such tree structures. Additional details regarding the generative model and the inference scheme are provided in the Supplementary Material.

## 4. Experiments

In this section we verify the effectiveness of the attribute tree process by applying it to a dataset which includes annotation for attributes. We consider the PASCAL VOC 2008 dataset, which includes bounding boxes for 20 categories and annotation for 64 attributes that were collected in Farhadi et al. (2009). The partitioning into training and testing sets as well as the attribute annotations and low-level image features are available online^1^. Each of the training and testing sets contains over 6000 instances from the 20 object classes: person, bird, cat, cow, dog, horse, sheep, airplane, bicycle, boat, bus, car, motorcycle, train, bottle, chair, dining-table, potted-plant, sofa, and tv/monitor. We defined 24 attributes in addition to those that were used in Farhadi et al. (2009): “pet,” “vehicle,” “alive,” “animal,” and the remaining 20 attributes were identical to the object categories. The annotation for the first four additional attributes was inferred from the object classes.

**Figure 2:**
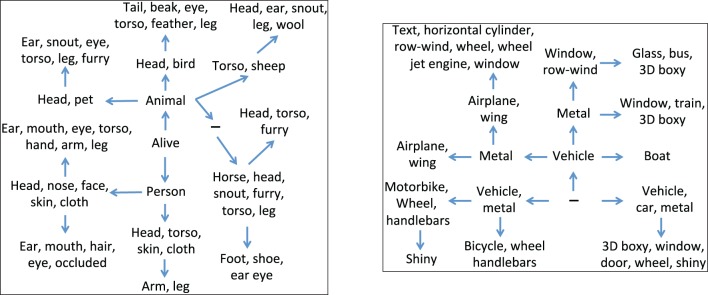
**Two fragments of the hierarchy learned using our generative model, using the annotated attributes available for the training set of the PASCAL dataset**. The left panel corresponds to the “living things” domain, whereas the right panel corresponds to the “transportation” domain. The sign (−) denotes an internal node.

In the first experiment, we ran our system to determine a hierarchy when using the ground truth attribute annotation of the training set as the observations, and show fragments of the hierarchy in Figure 2. We use two filters to determine what attributes are shown for a node in the figure, first only attributes with probability (see Equation 6 in the Supplementary Material) larger than 0.7, and second only attributes that have not appeared at an ancestor. The two fragments can be described as pertaining to the “living things” and “transportation” domains. An important observation regarding the organization of the attributes to nodes is that more abstract semantic concepts are assigned to the topmost nodes in the hierarchy, whereas the attributes assigned to the leaf nodes relate to fine-grained semantic concepts rather than to domains. For example, in the “transportation” fragment the domain label “vehicle” is assigned to nodes which precede more category specific attributes, such as “glass” or “window.”

**Figure 3:**
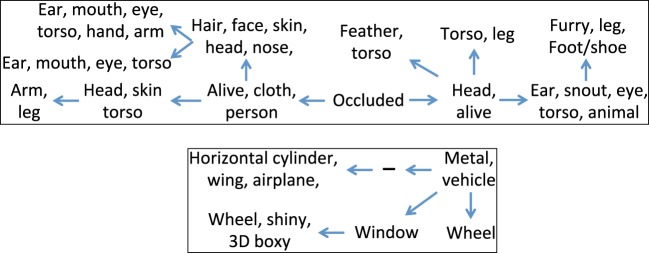
**Two fragments of the hierarchy learned using our generative model, using the attribute scores obtained for the testing set of the PASCAL dataset, when training the attribute detectors using the training set**. The top panel corresponds to the “living things” domain, whereas the bottom panel corresponds to the “transportation” domain. The sign (−) denotes an internal node.

In the second experiment, we used the low-level features and attribute annotation that are available for the training set, to train linear SVM classifiers to detect each of the 88 attributes. We then used these attribute classifiers to compute the attribute scores for each instance in the testing set. We ran our system to learn the hierarchy when using these attribute scores as the observations, and In Figure 3 we show the two fragments that correspond to the “living things” and “transportation” domains. As can be expected, due to the noisy nature of the attribute classifiers, the learned hierarchies are less descriptive as compared to those that were learned using the attribute annotations. However, they still reveal the super-subordinate relationship, and maintain a semantically coherent description for each node.

### 4.1. Evaluating the generative model as a hierarchical clustering algorithm

The attribute tree process model also provides us with a hierarchical clustering of the instances in the dataset, since it assigns each of them to a node in the tree. Therefore, we may consider comparing it to alternative hierarchical clustering algorithms, in order to evaluate its performance quantitatively. We consider two alternative approaches for hierarchical clustering: agglomerative hierarchical clustering (Jain and Dubes, 1988), and the factored Bernoulli likelihood model (Adams et al., 2010). Agglomerative hierarchical clustering uses an iterative bottom up approach to clustering. In the first iteration, each cluster includes a single data instance, and at each following iteration, the two clusters which are closest to each other are joined into a single cluster. This requires a distance metric, which measures the distance between clusters, to be defined. The algorithm concludes when the distance between the two farthest instances in each cluster is larger than some threshold. The factored Bernoulli likelihood model is a generative model that, similarly to our model, is based on the TSSBP. However, it uses a different generative process for obtaining the binary data instances.

In order to compare the performance of the different approaches quantitatively, we propose a new metric, which evaluates the degree to which the learned hierarchical clustering of the dataset accurately captures the ground truth semantic distance between the different categories. The semantic hierarchy provides us with a measure of the semantic distance between every two categories, in the form of the number of edges that separate them in the hierarchy. Our proposed metric, which we refer to as the average edge error, takes the form:

(4) $$\frac{2}{N(N-1)}\sum_{i\;=\;1}^{N-1}\sum_{j\;=\;i+1}^{N}|d_{H}(i,j)-d_{GT}(c(i),c(j))|,$$

where *c*(*i*) denotes the category of instance *i*, *N* denotes the number of image instances, *d*_*GT*_(*c*_1_, *c*_2_) denotes the number of edges separating categories *c*_1_ and *c*_2_ in the ground truth taxonomy of the categories, and *d*_*H*_(*i*, *j*) denotes the number of edges separating instances *i* and *j* in a hierarchy that is learned using a hierarchical clustering algorithm.

**Figure 4:**
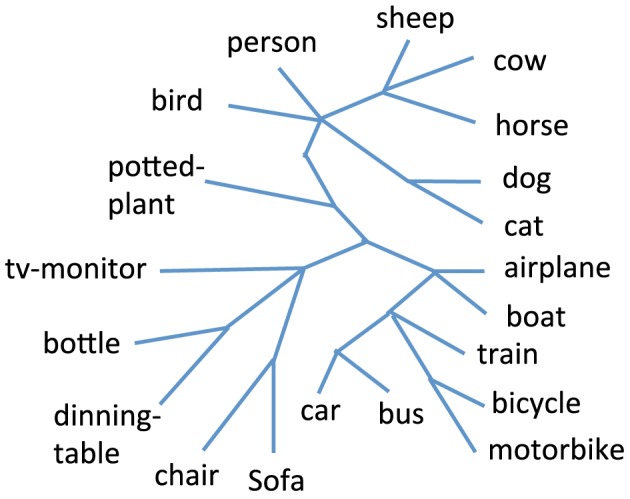
**The taxonomy for the 20 categories in the PASCAL dataset**.

**Figure 5:**
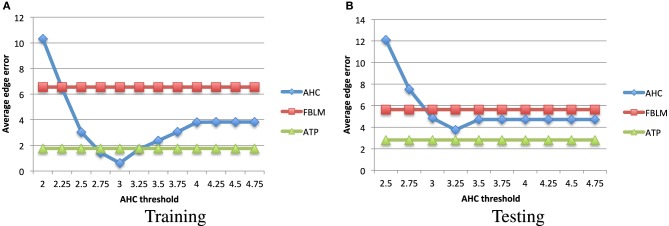
**The average edge error (Equation 4) vs. the agglomerative hierarchical clustering threshold parameter, for the hierarchical clustering obtained using the (A) training set's attribute annotations, and (B) attribute detectors applied to the testing set image instances**. Smaller values indicate better performance. It can be seen that our attribute tree process (ATP) algorithm outperforms the factored Bernoulli likelihood model (FBLM), and unlike the agglomerative hierarchical clustering (AHC), it is not as sensitive to the choice of the hyper-parameters.

In order to compute the average edge error for the PASCAL dataset, we use the taxonomy which is available in Binder et al. (2012) and is shown in Figure 4. We used the average distance metric for obtaining the hierarchical clustering using agglomerative hierarchical clustering. This algorithm is also known as the Unweighted Pair Group Method with Arithmetic Mean (Murtagh, 1984). We used the factored Bernoulli likelihood model implementation which is available online^2^. In Figure 5 we compare the average edge error for the attribute tree process (ATP), agglomerative hierarchical clustering (AHC), and factored Bernoulli likelihood model (FBLM), both when using the attribute annotation that is available for the training set, and when using the attribute scores obtained for the testing set, when training the attribute classifiers using the training set. Our implementation of agglomerative hierarchical clustering uses a threshold parameter that defines the maximum allowed Euclidean distance between two instances in each node, which effectively determines the number of nodes in the hierarchy. It can be seen that the performance of the agglomerative hierarchical clustering algorithm depends significantly on this threshold parameter. Furthermore our model outperforms the factored Bernoulli likelihood model.

#### 4.1.1. Sensitivity to hyper-parameters

In this work we used a uniform prior for the parameter *c*^(*d*)^, *d* = 1, …, *D* in Equation (3), such that *c*^(*d*)^ ~ *U*[l, *u*] with ℓ = 20, and *u* = 100. We also used the hyper-parameter values *a* = 10, and *b* = 5 in Equation (2). In order to evaluate the sensitivity of the attribute tree process to the choice of the hyper-parameters *a* and *b*, we compare in Table 1 the average edge error obtained using the annotation of the training set, when using different values for the hyper-parameters *a*, and *b*. It can be seen that when comparing to agglomerative hierarchical clustering in Figure 5, the attribute tree process is significantly less sensitive to the choice of hyper-parameters. When comparing to the factored Bernoulli likelihood model, even for the worst choice of the hyper-parameters the average edge error is still significantly better.

**Table 1:** **Average edge error using the attribute annotation of the training set, for different hyper-parameters**.

***a***	***b***	**Average edge error**
1	10	1.97
5	5	1.93
10	5	1.76
10	10	1.585
10	20	1.569

## 5. Discussion

We presented a new Bayesian non-parametric model, which we refer to as the attribute tree process, for learning domain hierarchies based on a semantic feature space. Such hierarchies have been shown to be necessary for tackling fine-grained visual recognition problems, in which the category count approaches the human capacity. Our model accounts for several important properties, such as capturing the inherent super-subordinate structure of the domains and concepts, accounting for uncertainty in the attribute observations, and maintaining a coherent semantic interpretation for each node. We also evaluated the attribute tree process as a hierarchical clustering algorithm, and demonstrated that it better captures the semantic distance between categories, as compared to alternative approaches, such as agglomerative hierarchical clustering, and the factored Bernoulli likelihood model. It is our belief that continued effort to develop computational models, both for learning the underlying semantic feature space as well the hierarchical organization of the domains, is necessary in order to better understand the corresponding mechanisms in the human visual system, as well as improve the performance of computerized visual recognition systems.

### Conflict of interest statement

The authors declare that the research was conducted in the absence of any commercial or financial relationships that could be construed as a potential conflict of interest.

## Footnotes

^1^http://vision.cs.uiuc.edu/attributes/

^2^http://hips.seas.harvard.edu/content/tree-structured-stick-breaking-hierarchical-data
