# Supplementary material for: A Bayesian generative model for learning semantic hierarchies
Source: Front Psychol. 2014 May 20;5:417. doi: 10.3389/fpsyg.2014.00417 (PMC4033064; doi:10.3389/fpsyg.2014.00417)
Supplement: Supplementary file 1 [file DataSheet1.PDF]

Frontiers in Psychology

Hypothesis and Theory
12 May 2014

Supplementary Material: A Bayesian Generative Model for Learning Semantic Hierarchies

Roni Mittelman [1,*], Min Sun [2], Benjamin Kuipers [1], and Silvio Savarese [3]

[1] *University of Michigan, Department of Electrical Engineering and Computer Science, Ann Arbor , MI, USA*
[2] *University of Washington, Department of Computer Science, Seattle WA, USA*
[3] *Stanford University, Department of Computer Science, Stanford CA, USA*

Correspondence*:
Roni Mittelman
University of Michigan, Department of Electrical Engineering and Computer Science, Ann Arbor , MI, USA, rmittelm@umich.edu

1 SUPPLEMENTARY MATERIAL

Here we provide additional details regarding our generative model, and the inference and learning schemes which we used.

1.1 TREE STRUCTURED STICK BREAKING PROCESS

The TSSBP is an infinite mixture model, where each mixture component is in one-to-one correspondence with a single node in an infinitely branching and infinitely deep tree. Let $\mathcal{T}$ denote the infinite set of node indices, and let π_ϵ denote the corresponding weight of the mixture component associated with node $\epsilon \in \mathcal{T}$, then one can sample a node z in the tree using

$$z \sim \sum_{\epsilon \in \mathcal{T}} \pi_\epsilon \delta_\epsilon(z), \tag{1}$$

where $\delta_\epsilon()$ denotes a Dirac delta function at ϵ.

Since the cardinality of the set $\mathcal{T}$ is unbounded, sampling from (1) is not trivial, however, an efficient sampling scheme was presented in [1]. Similarly, an efficient scheme for sampling from the posterior of $\{\pi_\epsilon\}_{\epsilon \in \mathcal{T}}$ given the node assignments of all the data instances, was also developed in [1].

1.2 THE ATTRIBUTE TREE PROCESS GENERATIVE MODEL

A node $z_i = \epsilon$ is sampled using the TSSBP, and the binary vector $[y_i^{(1)}, \ldots, y_i^{(D)}]^T$, which is associated with a data instance i that is assigned to node ϵ, is generated using the following scheme:

1. For each $\epsilon' \in \mathrm{A}(\epsilon) \cup \epsilon$, draw $\xi_{\epsilon',i}^{(d)} \sim \text{Bernoulli}(\theta_{\epsilon'}^{(d)})$, $d = 1, \ldots, D$,
2. Set $y_i^{(d)} = \bigoplus_{\epsilon' \in \mathrm{A}(\epsilon) \cup \epsilon} \xi_{\epsilon',i}^{(d)}$, $d = 1, \ldots, D$,

where $\bigoplus$ denotes the logical or operation, and $\mathrm{A}(\epsilon)$ denotes the set composed of all the ancestors of node ϵ. By marginalizing with respect to $\xi_{\epsilon',i}^{(d)}$, we can obtain a simplified scheme for generating $y_i^{(d)}$, $d = 1, \ldots, D$: first set

$$h_\epsilon^{(d)} = 1 - \prod\nolimits_{\epsilon' \in \mathrm{A}(\epsilon) \cup \epsilon} (1 - \theta_{\epsilon'}^{(d)}), \tag{2}$$

and then sample $y_i^{(d)} \sim \mathrm{Bernoulli}(h_\epsilon^{(d)})$ for every $d = 1, \ldots, D$.

The attribute vector x_i is obtained by flipping each element of the auxiliary random vector y_i with an appropriate probability $\omega^{(d)}$. By marginalizing over $y_i^{(d)}$, we have that the probably distribution of a data instance x_i takes the form:

$$p(x_i^{(d)} = 1|-) = 1 - ((1 - h_{z_i}^{(d)})(1 - \omega^{(d)}) + h_{z_i}^{(d)} \omega^{(d)}). \tag{3}$$

1.3 INFERENCE AND LEARNING

Inference is based on a Gibbs sampling scheme. In order to sample from the posterior of the node parameter $\theta_\epsilon^{(d)}$, we note that

$$\begin{aligned} &p(\theta_\epsilon^{(d)}|-) \propto \\ &\prod_{\epsilon' \in \epsilon \cup \mathrm{D}(\epsilon)} (1 - ((1 - h_{\epsilon'}^{(d)})(1 - \omega^{(d)}) + h_{\epsilon'}^{(d)} \omega^{(d)}))^{n_{\epsilon'}^{(1,d)}} \times ((1 - h_{\epsilon'}^{(d)})(1 - \omega^{(d)}) + h_{\epsilon'}^{(d)} \omega^{(d)})^{n_{\epsilon'}^{(0,d)}} \\ &\times \prod_{\epsilon'' \in \mathrm{Ch}(\epsilon)} \mathrm{Beta}(\theta_{\epsilon''}^{(d)}; c^{(d)} \theta_\epsilon^{(d)}, c^{(d)} (1 - \theta_\epsilon^{(d)})) \times \mathrm{Beta}(\theta_\epsilon^{(d)}; a_\epsilon^{(d)}, b_\epsilon^{(d)}), \end{aligned} \tag{4}$$

where $n_\epsilon^{(j,d)} = \sum_{i|z_i=\epsilon} \delta_j(x_i^{(d)})$ for $j = 0, 1$, $\mathrm{D}(\epsilon)$ denotes the set composed of all the descendants of node ϵ, $\mathrm{Ch}(\epsilon)$ denotes the child nodes of node ϵ, and $a_\epsilon^{(d)} = a/D$, $b_\epsilon^{(d)} = b(D-1)/D$, for $\epsilon = 0$ (the root node), and for any other node: $a_\epsilon^{(d)} = c^{(d)} \theta_{\mathrm{Pa}(\epsilon)}^{(d)}$, $b_\epsilon^{(d)} = c^{(d)}(1 - \theta_{\mathrm{Pa}(\epsilon)}^{(d)})$. The expression in (4) is a highly complicated function of $\theta_\epsilon^{(d)}$, and therefore we use slice-sampling [2] in order to sample from the posterior. The slice-sampler is very much a "black-box" algorithm, which only requires the log likelihood of (4) and very few parameters, and returns a sample from the posterior. We sample the node parameters using a two-pass approach, starting from the leaf nodes and moving up to the root, and subsequently moving down the tree from the root to the leaves.

In order to sample from $\omega^{(d)}$, we first sample the binary auxiliary random variables $y_i^{(d)}$ using

$$p(y_i^{(d)} = j|-) \propto p(y_i^{(d)} = j|-)(\delta_j(x_i^{(d)})(1 - \omega^{(d)}) + \delta_{1-j}(x_i^{(d)})\omega^{(d)}),\ j = 0, 1, \tag{5}$$

and then sample $\omega^{(d)}$ using

$$\omega^{(d)}|- \sim \mathrm{Beta}\big(\rho_0 + \sum_{i=1}^N \delta_1(y_i^{(d)} \mathrm{xor}\ x_i^{(d)}), \rho_1 + \sum_{i=1}^N \delta_0(y_i^{(d)} \mathrm{xor}\ x_i^{(d)})\big). \tag{6}$$

Sampling from the posterior of the parameter $c^{(d)}$ was also performed using slice sampling, by assuming a uniform prior over some interval. We note that slice sampling each of the parameters $\theta_\epsilon^{(d)}$ for $d = 1, \ldots, D$,

and each of $c^{(d)}$ for $d = 1, \ldots, D$, can be implemented in a parallel fashion. Therefore, the computational bottleneck in the attribute tree process is the number of nodes in the tree, rather than the number of attributes. Sampling from the posterior of the TSSBP parameters is performed using the algorithms developed in [1]. The parameters of the stick-breaking processes involved in the TSSBP construction are also learned from the data using slice-sampling, by assuming a uniform prior on some interval (as was also performed in [1]).

ACKNOWLEDGEMENT

We acknowledge the support of the NSF Grant CPS-0931474.

REFERENCES

[1]R. Adams, Z. Ghahramani, and M. I. Jordan. Tree-structured stick breaking for hierarchical data. In *Advances in Neural Information Processing Systems*, pages 19–27. 2010.
[2]R. Neal. Slice sampling. *Annals of Statistics*, 31:705–767, 2000.
